# Pulmonary Hypertension in Pulmonary Langerhans Cell Granulomatosis

**DOI:** 10.1155/2012/378467

**Published:** 2012-03-13

**Authors:** Matthias Held, Philipp Schnabel, Arne Warth, Berthold Jany

**Affiliations:** ^1^Medical Mission Hospital and Department of Internal Medicine, Academic Teaching Hospital, University of Würzburg, Salvatorstrasse 7, 97064 Würzburg, Germany; ^2^Institute of Pathology, Heidelberg University, Im Neuenheimer Feld 220, 69120 Heidelberg, Germany

## Abstract

*Introduction*. Pulmonary Langerhans cell granulomatosis is a rare disease with a variable course. In pulmonary Langerhans cell granulomatosis pulmonary hypertension is frequent and has an independent prognostic impact. A vasculopathy which ist not related to ventilatory disturbance and fibrosis has been identified. An arteriopathy and even a venulopathy have been described. Due to this possible venulopathy vasodilators carry a significant risk for pulmonary congestion and edema. No drugs have been approved until now. *Case Presentation*. One female with PLCG developed severe PH four years after primary diagnosis of pulmonary Langerhans cell granulomatosis. Retrospective analysis of lung biopsies revealed an arterial vasculopathy at the time of primary diagnosis without clinical signs of PH at this time. Sildenafil led to a sustained improvement of hemodynamic features and exercise capacity. *Conclusion*. This paper underlines that patients with PLCG with an arterial vasculopathy-related PH might improve under sildenafil. Further trials addressing treatment of PH and vasculopathy are needed.

## 1. Introduction

The course of adult pulmonary Langerhans cell granulomatosis (PLCG) is variable: in 25% the disease is spontaneously regressive, in 50% it remains stable, and in 25% there is a severe disease progression [1, 2]. In severe PLCG pulmonary hypertension (PH) is a common complication. In patients waiting for lung transplantation PH was found in 90% [3].

Crausman and Keogh showed that in patients with severe PLCG abnormalities of ventilatory function are common but not exercise-limiting. The impairment of exercise capacity is due to pulmonary vascular dysfunction [4, 5].

PH is often severe and not related to the ventilatory disorder or hypoxemia [3, 6, 7]. Different forms of a pulmonary vasculopathy affecting pulmonary arteries and pulmonary veins are described [6]. A progression of the vasculopathy in patients with no change of histopathological findings of lung parenchyma and a stable ventilatory disorder was reported [6]. The pathogenesis of this vasculopathy remains unclear: cytokines and growth factors may play an important role [8, 9].

Modern vasoactive drugs that are approved for pulmonary arterial hypertension (PAH) seem to be an attractive tool for treating PH caused by a pulmonary vasculopathy in PLCG, but data of randomized controlled trials are missing. A single experience of Bosentan was reported [10].

Side effects of a vasoactive therapy leading to pulmonary edema might be a possible complication in patients with a predominant venous vasculopathy [6].

## 2. Case Presentation

We report successful treatment of PH with sildenafil in a female patient with adult PLCG.

**Figure 1:**
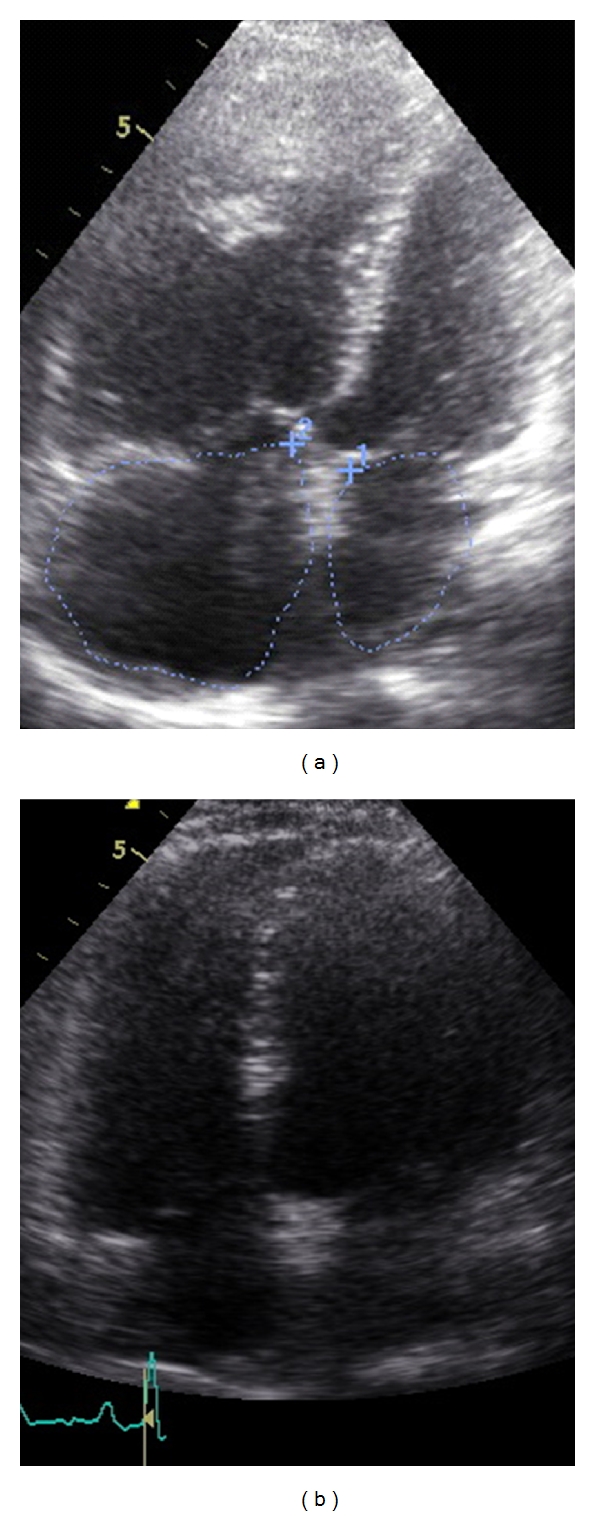
(a) Pulmonary Langerhans cell granulomatosis echocardiography, 4-chamber view, before treatment with sildenafil. (b) Pulmonary Langerhans cell granulomatosis echocardiography, 4-chamber view, after treatment with sildenafil.

**Figure 2:**
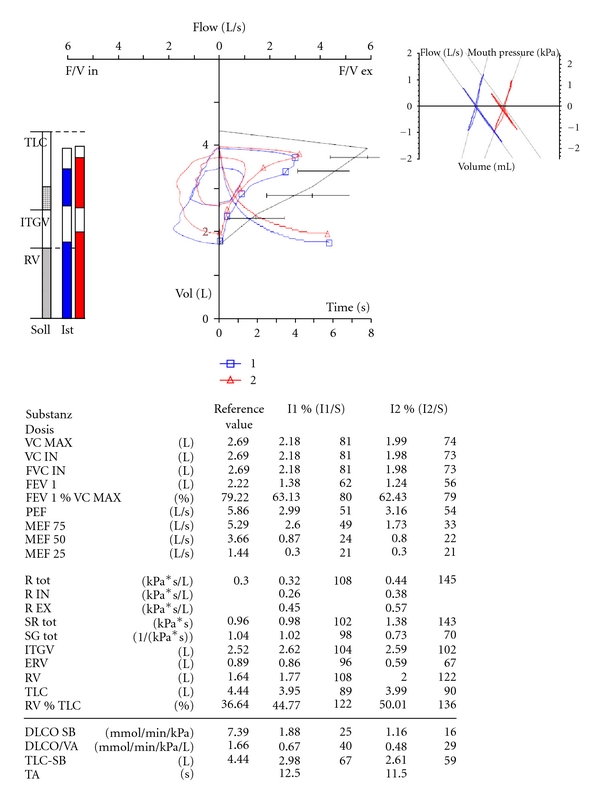
Pulmonary Langerhans cell granulomatosis, pulmonary function test.

**Figure 3:**
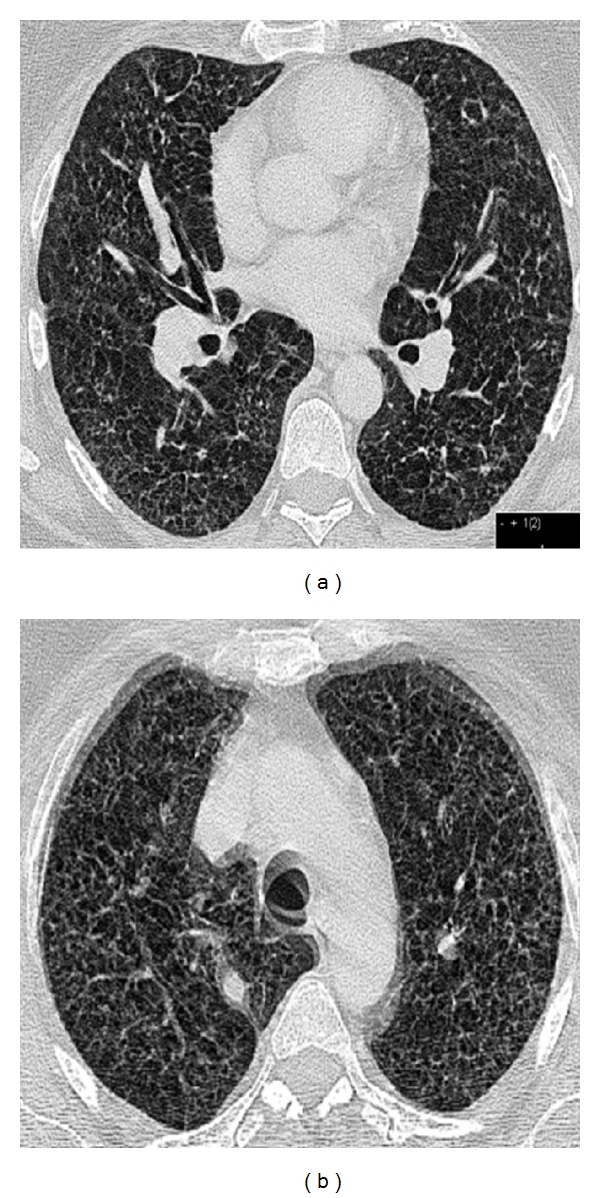
Pulmonary Langerhans cell granulomatosis, computed tomography.

**Figure 4:**
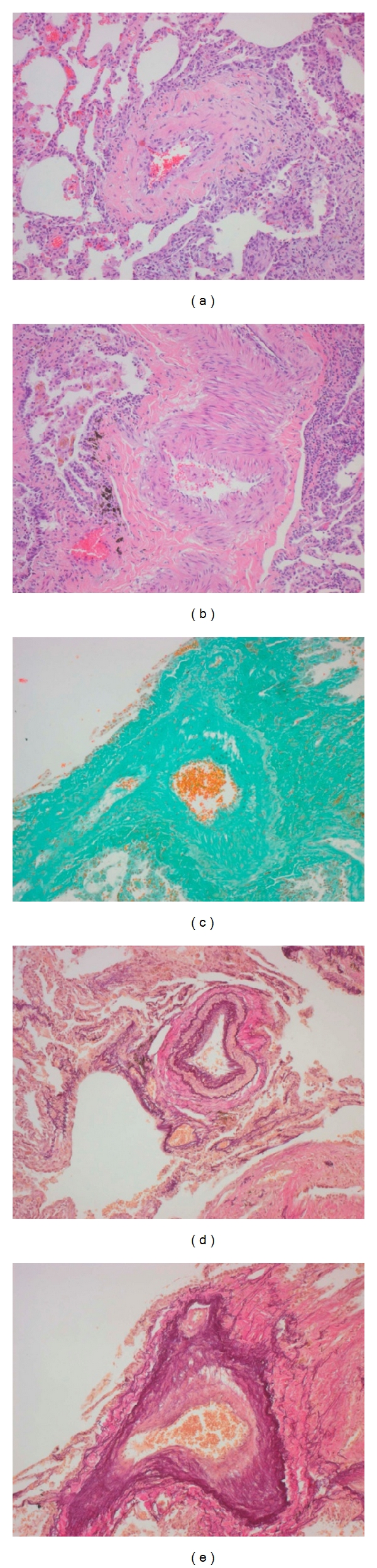
(a–e) Pulmonary Langerhans cell granulomatosis, pulmonary vessels, histological findings in 2005. (a, b) HE: Hämatoxylin-Eosin stain, (c) MG: Masson-Goldner-stain, and (d, e) EVG: Elastica van Gieson stain.

A 51-year-old female was first admitted to our PH outpatient clinic in March 2010. In 2005 a PLCG was diagnosed and proven by histopathological findings. In 2005 the patient was treated with corticosteroids. In 2010 the patient reported worsening of exercise tolerance and exertional dyspnea over the last months. Echocardiography showed elevated pulmonary artery pressure and dilated right atrium and right ventricle (Figure 1(a)). Pulmonary function test revealed moderate airflow obstruction and reduced transfer factor for carbon-monoxide (Figure 2). The computed tomography showed fibrosis, cysts, and bronchiectasis but no active lesions as noduli or ground glass opacities (Figure 3). We performed an RHC and so confirmed a severe precapillary PH (Table 1). Under exercise conditions the patient showed hypoxemia (Table 1). Retrospectively we analysed tissue samples of the year 2005 in which the PLCG was diagnosed: there were no clinical signs of PH, but tissue samples showed signs of a pulmonary vasculopathy (Figures 4(a)–4(e)). 

In March 2010 we first started prednisolone with 0.5 mg/kg. There was no effect on exercise capacity, lung function, oxygen partial pressure, radiological findings, or pulmonary artery pressure. After the start of sildenafil (20 mg three times a day) dramatic hemodynamic and clinical improvements were seen. Table 1 shows the results from RHC, 6-minute walking test, cardiopulmonary exercise testing, and echocardiography before and after initiation of sildenafil. Oxygen tension remained stable, and no pulmonary congestion was seen. In the following 12 months the patient remained stable, a rehabilitation was initiated, and listing for lung transplantation was considered.

## 3. Discussion

Not surprisingly there was no effect of a second course of corticosteroids in 2010 because CT scans showed endstage pulmonary Langerhans' cell granulomatosis with bronchiectasis and fibrosis without signs of activity as nodules.

The noted improvement under sildenafil is attributed to an effect on a pulmonary vasculopathy [6]. Surprisingly histopathological features of a vasculopathy had been already found in our patient 5 years ago, when she presented the first time with pulmonary Langerhans cell granulomatosis but without clinical signs of PH. As mentioned above foregoing studies reported arterial and venous vasculopathy. So a treatment with pulmonary vasodilators influencing the arterial vasculopathy was taken into account. On the other hand in patients with predominant venous vasculopathy side effects as pulmonary congestion caused by a vasodilatation of pulmonary arteries might appear and just have been described [6]. Such side effects did not appear under treatment with sildenafil in the patient reported here because the patient had a predominant arterial vasculopathy. Gas exchange was stable, and therapy was save.

The ventilatory disturbance and fibrosis and the underlying PLCG are not influenced by sildenafil, so that it is not surprising that the patient did not experience complete normalization of exercise capacity. For further management a rehabilitation was initiated and listing for lung transplantation was planned.

## 4. Conclusion

In PLCG and severe PH sildenafil is a promising option and should be considered for bridging to lung transplantation. Sildenafil should not be used uncritically and patients should be controlled carefully, because, under special circumstances, especially a predominant pulmonary venous vasculopathy side effects as pulmonary edema might appear.

PH due to PLCG belongs to group 5 of the Dana Point classification, no drugs are approved for this kind of PH. There is a need for further studies addressing treatment of PH and vasculopathy.

## Figures and Tables

**Table 1:** Pulmonary Langerhans cell granulomatosis: functional parameters before and under sildenafil.

Parameter	Before Sildenafil	3 months after the start of sildenafil	6 months after the start of sildenafil	9 months after the start of sildenafil
6-MWD (m)	350	400	380	420

Exercise Capacity (W)	36	65	55	55
VE/VCO_2_	80	41	36	33
EQO_2_ AT	40	38	32	33
EQCO_2_ AT	52	42	36	38

nT-pro-BNP pg/mL	301	54	22	16
PO_2_ (mmHg)	68	65	68	78
pCO_2_ (mmHg)	31	31	34	34
SO_2_%	94	94	94	96

TAPSE (mm)	18	19	18	24
Tei-Index	0,67	0,40	0,81	0,02
LVEI	1,22	1,0		1,0

mPAP (mmHg)	56	36	36	33
PVR dyn*s*cm^−5^	1035	500	496	338
PCWP (mmHg)	15	8	12	13
RAP (mmHg)	5	12	11	10
CI (l/min/m^2^)	2,0	2,7	2,8	2,6

## Abbreviations

CI: Cardiac index
cm: Centimeter
CT: Computed tomography
EQCO_2_ AT: Breathing equivalent for carbondioxide at anaerobic threshold
EQO_2_ AT: Breathing equivalent for oxygen at anaerobic threshold
FEV1: Forced expiratory volume after 1 second
FVC: Forced vital capacity
L: Liter
LVEI: Left ventricular eccentricity index
m: Meter
min: Minute
mL: Milliliter
mm: Millimeter
mmHg: Millimeter mercury
mPAP: Mean pulmonary artery pressure
nT-pro-BNP: N-terminal-pro-brain natriuretic peptide
pg: Picogram
PAH: Pulmonary arterial hypertension
PH: Pulmonary hypertension
pCO_2_: Partial pressure of carbon dioxide
PCWP: Pulmonary capillary wedge pressure
pO_2_: Partial pressure of oxygen
PVR: Pulmonary vascular resistance
RA area: Right atrial area
RAP: Right atrial pressure
RHC: Right heart catheterization
RVSP: Right ventricular systolic pressure
PHLCG: Pulmonary Langerhans cell granulomatosis
sec: Second
SO_2_: Oxygen saturation
6-MWD: Six-minute walking distance
TAPSE: Tricuspid plane systolic excursion
TLC: Total lung capacity
TLCO-VA: Transfer factor carbon monoxide per alveolar volume
VE/VCO_2_: VE/VCO_2_ slope
W: Watt
WHO: World Health Organization
WHO-FC: World Health Organization functional class
% pred: % predicted.
